# Control of Behavioral Arousal and Defense by a Glutamatergic Midbrain-Amygdala Pathway in Mice

**DOI:** 10.3389/fnins.2022.850193

**Published:** 2022-04-18

**Authors:** Shang-Yi Chen, Jing Yao, Yu-Duan Hu, Hui-Yun Chen, Pei-Chang Liu, Wen-Feng Wang, Yu-Hang Zeng, Cong-Wen Zhuang, Shun-Xing Zeng, Yue-Ping Li, Liu-Yun Yang, Zi-Xuan Huang, Kai-Qi Huang, Zhen-Ting Lai, Yong-Huai Hu, Ping Cai, Li Chen, Siying Wu

**Affiliations:** ^1^Department of Epidemiology and Health Statistics, The School of Public Health, Fujian Medical University, Fuzhou, China; ^2^Fujian Province Key Laboratory of Environment and Health, The School of Public Health, Fujian Medical University, Fuzhou, China; ^3^Department of Anesthesiology, Fujian Medical University Union Hospital, Fuzhou, China; ^4^School of Basic Medicine, Fujian Medical University, Fuzhou, China; ^5^School of Clinical Medicine, Fujian Medical University, Fuzhou, China; ^6^Department of Pharmacology, School of Pharmacy, Fujian Medical University, Fuzhou, China

**Keywords:** ventral tegmental area, central nucleus of the amygdala, sleep-wake behavior, defensive behavior, autism spectrum disorders

## Abstract

In response to external threatening signals, animals evolve a series of defensive behaviors that depend on heightened arousal. It is believed that arousal and defensive behaviors are coordinately regulated by specific neurocircuits in the central nervous system. The ventral tegmental area (VTA) is a key structure located in the ventral midbrain of mice. The activity of VTA glutamatergic neurons has recently been shown to be closely related to sleep–wake behavior. However, the specific role of VTA glutamatergic neurons in sleep–wake regulation, associated physiological functions, and underlying neural circuits remain unclear. In the current study, using an optogenetic approach and synchronous polysomnographic recording, we demonstrated that selective activation of VTA glutamatergic neurons induced immediate transition from sleep to wakefulness and obviously increased the amount of wakefulness in mice. Furthermore, optogenetic activation of VTA glutamatergic neurons induced multiple defensive behaviors, including burrowing, fleeing, avoidance and hiding. Finally, viral-mediated anterograde activation revealed that projections from the VTA to the central nucleus of the amygdala (CeA) mediated the wake- and defense-promoting effects of VTA glutamatergic neurons. Collectively, our results illustrate that the glutamatergic VTA is a key neural substrate regulating wakefulness and defensive behaviors that controls these behaviors through its projection into the CeA. We further discuss the possibility that the glutamatergic VTA-CeA pathway may be involved in psychiatric diseases featuring with excessive defense.

## Introduction

In the face of threats, animals in nature exhibit a series of defensive behaviors that include avoidance, escape, and hiding. When animals are in a dangerous environment, they must maintain a higher state of wakefulness to enable timely defensive behavior to protect themselves from external threats. It is believed that sleep–wake behavior and defensive behaviors are coordinately regulated by specific neurocircuits in the central nervous system. The ventral tegmental area (VTA) is a key structure located in the ventral midbrain that contains dopaminergic, GABAergic, and glutamatergic neurons (Nair-Roberts et al., 2008). Previous studies have shown that the VTA is involved in regulation of multiple behaviors, including reward, motivation, learning, and aversion (Morales and Margolis, 2017; Yu et al., 2019). Recent results showed that the activity of VTA glutamatergic neurons is closely related to sleep–wake behavior. Recordings of calcium signals by fiber photometry demonstrated that VTA glutamatergic neurons had significantly increased activity during wakefulness and REM sleep, but lower activity during NREM sleep (Yu et al., 2019). Additionally, chronic lesioning of VTA glutamatergic neurons reduces the total amount of wakefulness and increases NREM sleep amount in active phase of mice (Yu et al., 2019). VTA glutamatergic neurons have also been shown to participate in the regulation of innate defensive behaviors (Barbano et al., 2020). The calcium signals of VTA glutamatergic neurons were significantly enhanced in response to threatening visual stimulation, as well as in response to exposure to the synthetic predator odor, trimethylthiazoline (Barbano et al., 2020). Genetic ablation of VTA glutamatergic neurons obviously influences escape behavior in response to threatening stimuli, as indicated by significantly decreased running numbers and prolonged escape latency in mice (Barbano et al., 2020). These results imply that VTA glutamatergic neurons play an important role in coordination of sleep–wake behavior and defensive behavior, but the precise role of glutamatergic VTA and its underlying neurocircuits remain unclear.

Neuroanatomical results have shown that glutamatergic VTA abundantly innervates the amygdala (Taylor et al., 2014). As a key part of the amygdala, the central nucleus of the amygdala (CeA) orchestrates a variety of behaviors, including sleep–wake behavior and defensive behaviors. *In vivo* electrophysiological results have shown that the majority of neurons in the CeA are related to sleep–wake behavior, and that these neurons fire slowly during NREM sleep and increase their discharge during wakefulness and/or REM sleep (Jha et al., 2005). Pharmacological inactivation of the CeA by microinjections of tetrodotoxin significantly decreased REM sleep and reduced arousal in the first hour, resulting in shortened NREM sleep latency and decreased locomotion in mice (Tang et al., 2005). The CeA has also long been known to be involved in defensive response, which is proven to mediate physiological and behavioral changes in the face of threat (Fadok et al., 2018; Gu et al., 2020; van den Burg and Hegoburu, 2020). Chemogenetic activation and inhibition of noradrenergic terminals in the CeA are sufficient for bidirectional modulation of defensive responses elicited by conditioned threats (Gu et al., 2020). Pharmacological manipulation of the CeA modulates the expression of innate defensive behavior induced by visual stimuli (Zhou et al., 2019). Collectively, these results suggest that the glutamatergic VTA-CeA pathway plays a key role in regulation of wakefulness and defensive behaviors.

In this study, we employed an optogenetic approach to selectively activate VTA glutamatergic neurons and investigated their roles in the regulation of defensive behaviors. Using synchronous polysomnographic recordings, we determined the effects of VTA glutamatergic activation on initiation and maintenance of wakefulness. Finally, we used viral-mediated anterograde activation to identify the downstream target of wake- and defense-promoting effects of VTA glutamatergic neurons, namely, the central nucleus of the amygdala (CeA). Collectively, our results illustrate that the glutamatergic VTA is a key neural substrate that regulates wakefulness and defensive behaviors through its projection into the CeA.

## Materials and Methods

### Animals

All of the experimental procedures were conducted according to the guidelines of the Ethics Committee of Laboratory Animal Management of Fujian Medical University. Adult male Vglut2-ires-Cre mice (catalog #016963, Jackson Laboratory) were used for the all studies. Mice were at least 8 weeks old and weighed at least 22 g. Sixteen Vglut2-Cre mice (ChR2 group, *n* = 8; mCherry group, *n* = 8) were used in optogenetic studies of VTA soma. Sixteen Vglut2-Cre mice (ChR2 group, *n* = 8; mCherry group, *n* = 8) were used in optogenetic studies of VTA-CeA pathway. Animals were housed under an automatic 12-h light/dark cycle. One week before the behavioral tests and polysomnography recording, mice were kept on a 12-h reverse light/dark cycle (lights at Zeitgeber Time 00:00 and off at Zeitgeber Time 12:00).

### Stereotaxic Surgery and Virus Injection

In this study, rAAV-EF1a-DIO-hChR2-mCherry and rAAV-EF1a-DIO-mCherry viruses were obtained from Taitool Bioscience (China) and BrainVTA (China). The titer of rAAV-EF1a-DIO-hChR2-mCherry and rAAV-EF1a-DIO-mCherry is 2.62 × 10^12^ and 5.20 × 10^12^ particles/mL, respectively. Surgery was conducted in a stereotaxic apparatus, 3 and 1% isoflurane was used for induction and maintenance of anesthesia, respectively. A craniotomy was performed following asepsis, after which 300 nL AAV viruses were unilaterally injected into the ventral tegmental area (coordinates: AP: −3.4 mm, ML: −0.2 mm, DV: −4.1 mm) and optical fibers were implanted on the ventral tegmental area (AP: −3.4 mm, ML: −0.2 mm, DV: −3.7 mm) or the central amygdala (AP: −1.25 mm, ML: ±2.5 mm, DV: −4.55 mm). A glass pipette was used for the viral injection, and the injecting speed was controlled at a rate of 50 nL/min. After injection, the glass pipette was left in the injection site for 10 min to allow the virus to spread. The body temperature of the mice was controlled at 36 ± 0.5°C throughout the anesthesia procedure using an automated temperature control system. Following surgery, the mice were moved to an incubator until they fully recovered.

### Behavioral Tests

#### Burrowing Tests

Burrowing is a typical defensive behavior (Blanchard and Blanchard, 1989; Kitaoka, 1994). In the burrowing experiment, mice were housed individually in a home-cage apparatus for at least 1 day before the experiment with *ad libitum* access to water and food. The experiment consisted of two 1-min tests. During the first minute, the animals received no light stimulation, while in the second minute, blue light (473 nm, 10 ms, 20 Hz) was delivered to the VTA or CeA *via* the optical fiber. The burrowing time was then calculated by a blind experimenter.

#### Open Field Test

In the open field test, mice were placed in a cube arena (60 cm × 60 cm × 60 cm) that they were allowed to explore freely for at least 10 min before the experiment. The test consisted of three 5-min sessions (pre-test period, stimulation period, and post-test period). Blue light (473 nm, 10 ms, 10 Hz) was delivered to the target brain region intermittently (3 s-on, 2 s -off).

#### Real-Time Position Preference Test

The Real-time position preference (RTPP) apparatus is comprised of two distinct chambers, black chamber and white chamber (L × W × H = 20 cm × 20 cm × 30 cm), with a 6-cm-wide opening in the middle, the design of which is modified based on the previous study (Wang et al., 2015; Cai et al., 2022). This test included two 10-min sessions (pre-test period and stimulation period). During the acclimation of RTPP apparatus, mice were allowed to freely explore the apparatus for 10 min. All of the tested mice preferred to stay in the black chamber without light stimulation; therefore, this chamber was defined as the stimulation chamber. During the 10-min stimulation period, blue light (473 nm, 10 ms, 10 Hz) was delivered once the animals entered the stimulation chamber, while the light stimulation was suspended immediately after the mice left the stimulation chamber.

#### Hiding Box Test

We adapted a hiding apparatus developed in previous studies to examine animals’ hiding behavior (Wang et al., 2015; Chen et al., 2020). The hiding apparatus was a plexiglass cuboid with an open area, hiding box, and feeding area. The hiding box and feeding area were placed on the diagonal corner of the hiding box. After mice were acclimated to the apparatus for 12 h, standard food and 2–3 peanuts were placed in the feeding area. After mice entered the feeding area and fed for about 10 s, blue light (473 nm, 10 ms, 0 Hz, 5 Hz, 10 Hz, and 20 Hz) was transmitted to the target brain region of the mice through the optical fiber. Light stimulation was turned off when the mice returned to the hiding chamber.

### Polysomnographic Recordings and Analysis

For polysomnographic recording, mice were implanted with both electroencephalogram (EEG) and electromyogram (EMG) electrodes. Two stainless steel screws were inserted through the skull of the cortex and served as EEG electrodes, and two stainless-steel Teflon-coated wires were bilaterally placed in bilateral trapezius muscles and served as EMG electrodes, as described previously (Cai et al., 2020, 2022). One week before starting the recording, the mice were transferred to a transparent cylinder and housed individually. At least 3 days before the recording, the EEG/EMG electrodes were connected to a cable line. A slip-ring was used to connect the recording cable and ensure that mice could move freely. EEG/EMG signals were amplified, filtered and recorded using the Vital Recorder Software (Kissei Comtec, Japan). In acute optogenetic stimulation experiments, a light source emitted from a laser diode (Newdoon, China) was transmitted *via* a rotating optical joint (FRJ_FC-FC, Doric Lenses, Canada) for optogenetic stimulation. Blue light (473 nm) and yellow light (589 nm) were applied from 5 to 40 Hz for 16 s to examine the NREM-WAKE transition probability, and 60 s of continuous stimulation was applied to test their NREM-WAKE transition latency. For the chronic light stimulation experiment of the VTA glutamatergic neurons, we administered light stimulation at Zeitgeber Time 02:00–05:00, while we applied light stimulation on the glutamatergic VTA-CeA pathway at Zeitgeber Time 02:00–03:00. Sleep–wake states were automatically classified into wake, NREM and REM sleep according to the SleepSign software criteria. After analyzed by software, all epochs were further checked and corrected artificially. All of the experiments were conducted during the inactive period of mice (lights on period, Zeitgeber Time 00:00–12:00).

### Immunofluorescence

The mice were deeply anesthetized with sodium pentobarbital. The whole brain was fixed with 4% paraformaldehyde (PFA) following perfusion with PBS, then it was subjected to gradient dehydration by immersion in 20 and 30% sucrose. The brain was then cut into 20-μm sections using a freezing microtome (CM1950, Leica) and washed in 0.01 M PBS for 5 min to remove the embedded OCT (catalog #4583, Sakura). We then perforated the membrane with 0.7% Triton x-100 and incubated slices with rabbit-anti-c-fos antibody (1:1000, #Ab190289, Abcam) for 24 h. Finally, the slices were incubated in goat anti-rabbit 488 antibody (1:1000, catalog No. #111-545-003, Jackson) at 37°C for 2 h, after which they were subjected to 5 min of DAPI staining and scanned using a fluorescence microscope (DMi8, Leica).

### Statistical Analysis

GraphPad 8.0 and SPSS 20.0 were used to process all data. Student’s *t*-test, the Kaplan–Meier method, or two-way repeated measure ANOVA analyses were conducted following Bonferroni’s *post hoc* test. All data were expressed as the Mean ± SEM. In all cases, *P* < 0.05 was considered to be statistically significant.

## Results

### Photoactivation of Ventral Tegmental Area Glutamatergic Neurons Initiates and Maintains Wakefulness

**FIGURE 1:**
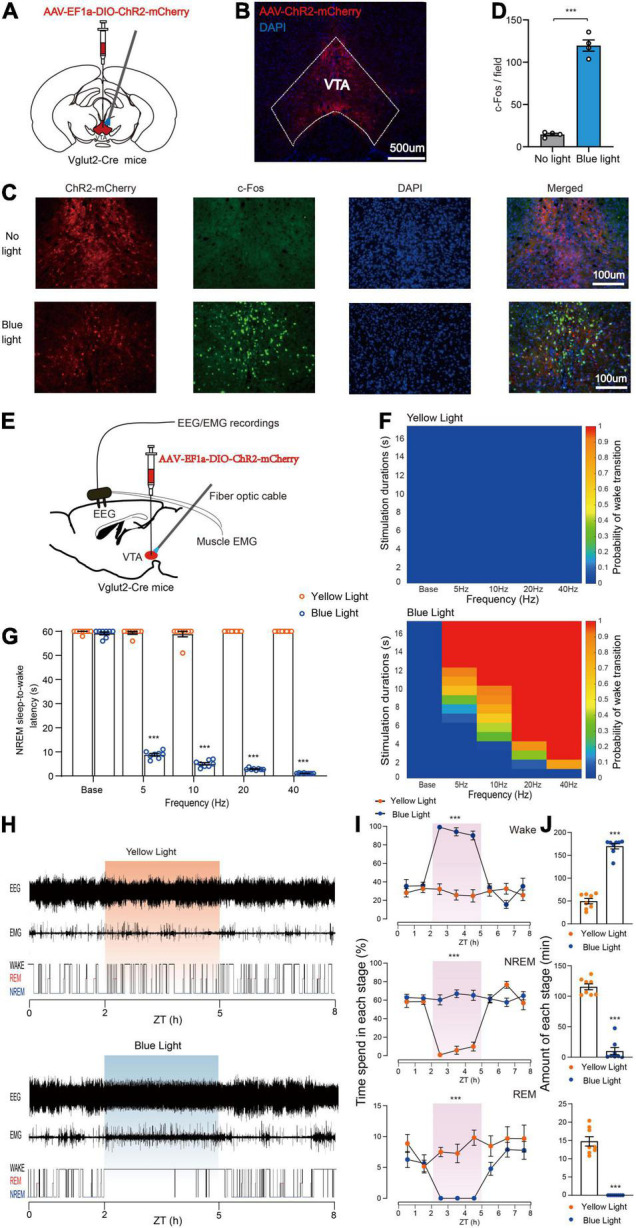
Photoactivation of VTA glutamatergic neurons initiates and maintains wakefulness. **(A)** Schematic of AAV-EF1a-DIO-ChR2-mCherry expression in the VTA. **(B)** Representative fluorescent image showing restrictive expression of ChR2-mCherry in the VTA area. **(C)** Fluorescent images showing c-Fos expression in the VTA without (top) or with blue light stimulation. **(D)** Quantitative analyses of c-Fos expression (*n* = 4, 8 sections for each group. Two-tailed unpaired *t*-test; *t*_6_ = 15.50, *P* < 0.001). **(E)** Diagram of photostimulation of VTA glutamatergic neurons. **(F)** Heat maps showing the probability of NREM-Wake transition. **(G)** Latencies of NREM-Wake transition upon stimulation with different frequencies of light (*n* = 8 per group, paired *t*-test; Base, *t*_7_ = 1.187, *P* = 0.2741; 5 Hz, *t*_7_ = 90.60, *P* < 0.001; 10 Hz, *t*_7_ = 44.12, *P* < 0.001; 20 Hz, *t*_7_ = 397.1, *P* < 0.001; 40 Hz, *t*_7_ = 1200, *P* < 0.001). **(H)** Example of EEG/EMG traces showing the effects of light stimulation of VTA glutamatergic neurons on sleep–wake behaviors. **(I)** Time course change in sleep–wake behaviors during long-term photostimulation of VTA glutamatergic neurons during ZT 02:00–05:00 [*n* = 8, two-way repeated-measures ANOVA; wake *F*(1,14) = 51.74, *P* < 0.001; NREM *F*(1,14) = 50.06, *P* < 0.001; REM *F*(1,14) = 23.66, *P* < 0.001]. **(J)** Sleep and wake amount during 3-h long-term light stimulation (*n* = 8, paired *t*-test; wake *t*_7_ = 17.93, *P* < 0.001; NREM *t*_7_ = 15.98, *P* < 0.001; REM *t*_7_ = 11.47, *P* < 0.001). Data are expressed as the mean ± SEM. ***P < 0.001.

To determine the specific role of VTA glutamatergic neurons in sleep–wake regulation, we tested the effects of activating VTA glutamatergic neurons using an optogenetic approach. Adeno-associated virus (AAV) encoding EF1a-DIO-ChR2-mCherry was injected into the VTA of Vglut2-Cre mice, and optical fiber was implanted above the VTA to selectively activate VTA glutamatergic neurons (Figure 1A). Electrodes were implanted in the cerebral cortex and posterior cervical muscle of mice to enable simultaneous recording of EEG/EMG signals (Figure 1E). At 4 weeks after AAV transfection, strong expression of ChR2-mCherry was observed in the VTA (Figure 1B). To further certify that ChR2-mCherry neurons were activated by photostimulation, blue light (473 nm) was delivered to the VTA through the optical fiber, and expression of c-Fos protein was checked. We found blue light stimulation drove a higher level of c-Fos expression in the VTA compared with no light stimulation (Figures 1C,D), indicating that VTA glutamatergic neurons are potentially activated by photoactivation. At 4 weeks after AAV transfection, mice were adapted in the recording chamber for about 7 days, and EEG/EMG signals of mice were recorded by a polygraphic recording system (Figure 1E).

To examine the effects of photoactivating VTA glutamatergic neurons on the probability of NREM-to-wake transition, short-term blue or yellow (control) light stimulations were delivered to the VTA. We found that blue light stimulation significantly increased the probability of transition from non-rapid eye movement (NREM) sleep to wakefulness compared with the yellow light stimulation (Figure 1F). Our results showed that blue light stimulation at higher frequencies (10, 20, and 40 Hz) strongly increased the probability of NREM-to-wake transition (Figure 1F). Photostimulation at 20 Hz potently changed brain state and increased the probability of wakefulness (Supplementary Figure 1). Blue, but not yellow, light stimulation increased the probability of transition in a frequency-dependent fashion (Figure 1F). Next, the effects of VTA glutamatergic activation on the latency of NREM-to-wake transition was checked. Our results showed that blue light stimulation significantly decreased the latency compared with yellow light stimulation; and the latency gradually decreased as the blue light stimulation frequency increased (Base, 59.8 ± 0.3 s at yellow light stimulation vs. 59.2 ± 0.6 s at blue light stimulation, *n* = 8, *P* = 0.2741; 5 Hz, 59.5 ± 0.5 s at yellow light stimulation vs. 8.8 ± 0.5 s at blue light stimulation, *n* = 8, *P* < 0.001; 10 Hz, 58.9 ± 1.1 s at yellow light stimulation vs. 5.1 ± 0.5 s at blue light stimulation, *n* = 8, *P* < 0.001; 20 Hz, 60.0 ± 0.0 s at yellow light stimulation vs. 2.9 ± 0.1 s at blue light stimulation, *n* = 8, *P* < 0.001; 40 Hz, 60.0 ± 0.0 s at yellow light stimulation vs. 1.1 ± 0.1 s at blue light stimulation, *n* = 8, *P* < 0.001, paired *t*-test, Figure 1G). Blue light stimulation at 40 Hz induced an immediate NREM-to-wake transition with an average latency of about 1.1 s (Supplementary Video 1). These results illustrated that activation of VTA glutamatergic neurons is sufficient to initiate wakefulness.

Next, we examined the effects of photoactivating VTA glutamatergic neurons in the maintenance of wakefulness. Long-term light stimulation (10 ms, 10 Hz, 20 s-on 40 s-off, 3 h) was delivered during the light period when mice were inactive (Zeitgeber Time 02:00–05:00). Long-term blue light stimulation markedly changed the sleep structure of mice, while long-term yellow light stimulation did not. The hypnograms indicated that mice woke from sleep and maintained wakefulness nearly for 3 h during the blue light stimulation period (Figure 1H). Moreover, the wakefulness of mice was significantly increased during the 3 h of blue light stimulation compared with yellow light stimulation (Figure 1I). The total amount of wakefulness was significantly increased during the light stimulation period, while the total amount of NREM sleep and REM sleep was significantly decreased (Wake, 49.7 ± 5.8 min at yellow light stimulation vs. 169.9 ± 5.9 min at blue light stimulation, *n* = 8, *P* < 0.001; NREM, 115.5 ± 4.8 min at yellow light stimulation vs. 10.0 ± 5.9 min at blue light stimulation, *n* = 8, *P* < 0.001; REM, 14.8 ± 1.3 min at yellow light stimulation vs. 0.0 ± 0.0 min at blue light stimulation, *n* = 8, *P* < 0.001, paired *t*-test, Figure 1J). Together, these results clearly demonstrate that activation of VTA glutamatergic neurons is sufficient to initiate and maintain wakefulness.

### Photoactivation of Ventral Tegmental Area Glutamatergic Neurons Promotes Burrowing and Fleeing Behaviors

**FIGURE 2:**
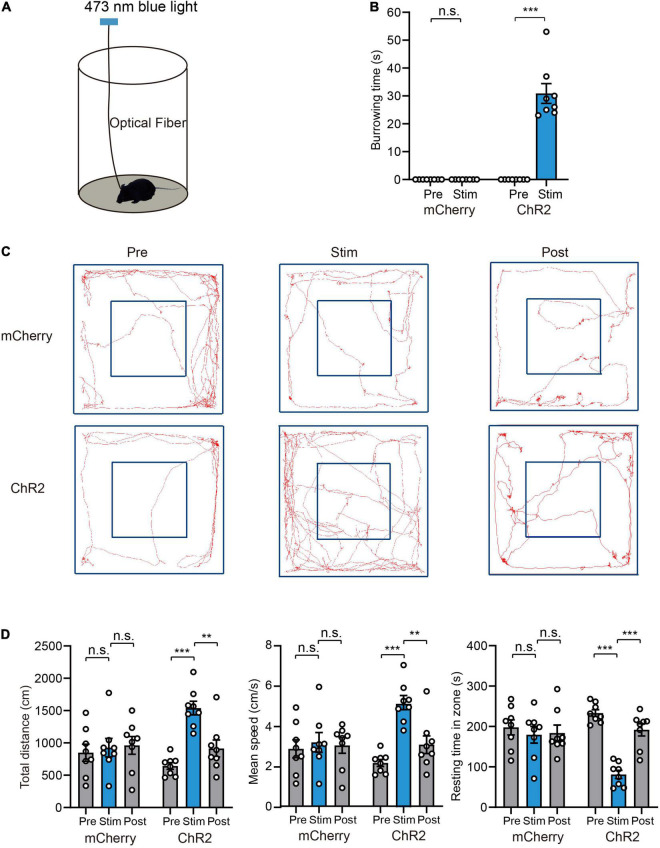
Photoactivation of VTA glutamatergic neurons promotes burrowing and fleeing behaviors. **(A)** Schematic of the home-cage test. **(B)** The total burrowing time upon stimulating VTA glutamatergic neurons in the home-cage test [*n* = 8 ChR2 vs. *n* = 8 mCherry; two-way repeated-measures ANOVA, virus × stimulation, *F*(1,14) = 76.38, *P* < 0.001, Bonferroni *post hoc* comparison]. **(C)** Examples of tracking traces of mice in open field tests. **(D)** Photostimulation of VTA glutamatergic neurons influenced total distance, mean speed and resting time in open field experiments [*n* = 8 ChR2 vs. *n* = 8 mCherry; two-way repeated-measures ANOVA, virus × stimulation, *F*(2,28) = 10.35, *P* = 0.004, *F*(2,28) = 9.747, *P* = 0.0006, *F*(2,28) = 13.70, *P* < 0.001, Bonferroni *post hoc* comparison]. Data are expressed as mean ± SEM. **P < 0.01, ***P < 0.001, n.s., not significant.

High arousal is required for defensive behaviors, and quick transition from sleep to wakefulness helps to avoid potential danger or approaching predators. Based on above results showing that activation of VTA glutamatergic neurons strongly promotes wakefulness, we hypothesized that VTA glutamatergic neurons are involved in the regulation of defensive behaviors. To check this, AAV encoding EF1a-DIO-ChR2-mCherry was microinjected into the VTA of Vglut2-Cre mice and optical fiber was implanted concomitantly above the VTA. At 4 weeks after AAV transfection, we examined the behavior change induced by photoactivating VTA glutamatergic neurons. We first evaluated defense-related behaviors in the animals’ home cages (Figure 2A). In the home-cage test, ChR2 group mice showed continuing burrowing behavior after the blue light was delivered (Supplementary Video 2). Blue light stimulation significantly increased the burrowing time in ChR2 group (Pre 0.0 ± 0.0 s vs. Stim 30.9 ± 3.5 s, *n* = 8, two-way repeated-measures ANOVA, *P* < 0.001, Figure 2B). In mCherry-control mice, blue light stimulation did not promote burrowing behavior (mCherry group: Pre 0.0 ± 0.0 s vs. Stim 0.0 ± 0.0 s, *n* = 8, two-way repeated-measures ANOVA, *P* > 0.05, Figure 2B).

Fleeing is a typical reaction to approaching predators. To evaluate the effects of light stimulation on fleeing behavior, we evaluated the performance of mice in an open field test. Light stimulation strongly increased the locomotion of mice (Figure 2C and Supplementary Video 3). Specifically, light stimulation significantly increased the total distance (mCherry group: Pre 848.8 ± 132.2 cm vs. Stim 926.4 ± 142.4 cm vs. Post 960.8 ± 137.8 cm, ChR2 group: Pre 643.1 ± 52.4 cm vs. Stim 1539.5 ± 104.5 cm vs. Post 914.0 ± 131.4 cm, *n* = 8, *P* = 0.004), mean velocity (mCherry group: Pre 2.8 ± 0.4 cm/s vs. Stim 3.2 ± 0.5 cm/s vs. Post 3.0 ± 0.4 cm/s, ChR2 group: Pre 2.1 ± 0.2 cm/s vs. Stim 5.1 ± 0.3 cm/s vs. Post 3.0 ± 0.4 cm/s, *n* = 8, *P* = 0.0006), and decreased resting time in the zone [mCherry group: Pre 198.0 ± 18.6 s vs. Stim 179.6 ± 20.6 s vs. Post 183.9 ± 19.6 s, ChR2 group: Pre 233.2 ± 8.5 s vs. Stim 81.3 ± 10.2 s vs. Post 191.8 ± 15.2 s, *n* = 8, two-way repeated-measures ANOVA, *F*(2,28) = 13.70, *P* < 0.001] (Figure 2D). In the mCherry-control mice, blue light stimulation did not change the locomotion of mice (Figures 2C,D).

### Photoactivation of Ventral Tegmental Area Glutamatergic Neurons Promotes Avoidance and Hiding Behaviors

**FIGURE 3:**
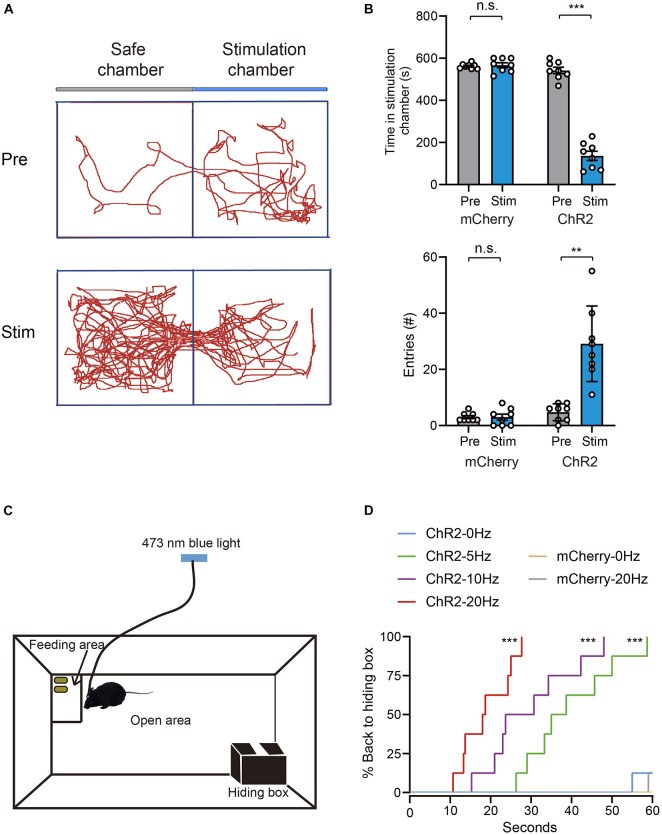
Photoactivation of VTA glutamatergic neurons promotes avoidance and hiding behavior. **(A)** Example trajectories of mice in RTPP tests. **(B)** Photostimulation of VTA glutamatergic neurons affected the time spent in the stimulation chamber and safe chamber entries of mice in the RTPP experiment [*n* = 8 ChR2 vs. *n* = 8 mCherry; two-way repeated-measures ANOVA, virus × stimulation, *F*(1,14) = 196.1, *P* < 0.001; *F*(1,14) = 27.82, *P* = 0.0001, Bonferroni *post hoc* comparison]. **(C)** Schematic of hiding box experiment. **(D)** Real-time probability of hiding upon stimulating glutamatergic VTA (*n* = 8 ChR2, *n* = 8 mCherry; Kaplan–Meier5 survival analysis with Bonferroni’s multiple comparison; Log rank = 80.77, *P* < 0.001, mCherry: 20 Hz-sham, *P* = 0.32; ChR2: 5 Hz-sham, *P* < 0.001; 10 Hz-sham, *P* < 0.001; 20 Hz-sham, *P* < 0.001). Data are represented as the mean ± SEM. **P < 0.01, ***P < 0.001, n.s., not significant.

Given that activation of VTA glutamatergic neurons increased burrowing and fleeing behaviors, we investigated whether these neurons mediate other defensive behaviors, such as avoidance. To determine whether VTA glutamatergic neurons mediate avoidance, we conducted a real-time place preference (RTPP) test as previously described (Wang et al., 2015; Cai et al., 2022). In this test, light stimulation was applied when mice came to the stimulation chamber and stopped immediately when they entered the safe chamber (Supplementary Video 4). Our results showed that blue light stimulation significantly decreased the time spent in stimulation chamber and increased the number of safe chamber entries in ChR2 group, but not in mCherry group [time in stimulation chamber, mCherry group: Pre 562.1 ± 4.4 s vs. Stim 566.9 ± 11.6 s, ChR2 group: Pre 541.9 ± 14.9 s vs. Stim 136.1 ± 22.1 s, *n* = 8, two-way repeated-measures ANOVA, *F*(1,14) = 196.1, *P* < 0.001; entries number, mCherry group: Pre 3.0 ± 0.5 vs. Stim 3.0 ± 1.1, ChR2 group: Pre 4.8 ± 1.1 vs. Stim 29.1 ± 4.8, *n* = 8, two-way repeated-measures ANOVA, *F*(1,14) = 27.82, *P* = 0.0001, Figures 3A,B].

Hiding is another effective means to evade predators. When encountering a predator, animals will hide in nearby nest or shelter if one is available. To test whether activation of VTA glutamatergic neurons can induce hiding, we adapted another apparatus to test mice’s hiding behavior according to previous studies (Wang et al., 2015; Chen et al., 2020). The hiding apparatus consisted of an open area at the center, a hiding box and a feeding area located in a diagonal corner (Figure 3C). Before the test, the mice were acclimated to the apparatus for at least 1 day. After the acclimation, peanuts were added to the feeding area to encourage continuous exploration of mice. After the mice entered the feeding area and stayed for about 10 s, blue light stimulation was administered. We found that, when 5 Hz blue light was administered, mice instantly stopped feeding and went back to their hiding box (Supplementary Video 5). Blue light stimulation frequency-dependently increased the probability of hiding and decreasing the latency of hiding (Figure 3D). Application of blue light to mCherry-control mice did not interrupt feeding behavior or induce hiding behavior (Figure 3D). These results clearly demonstrate that activation of VTA glutamatergic neurons is sufficient to induce avoidance and hiding behaviors.

### Photoactivation of Glutamatergic VTA-CeA Pathway Promotes Defensive Behaviors

**FIGURE 4:**
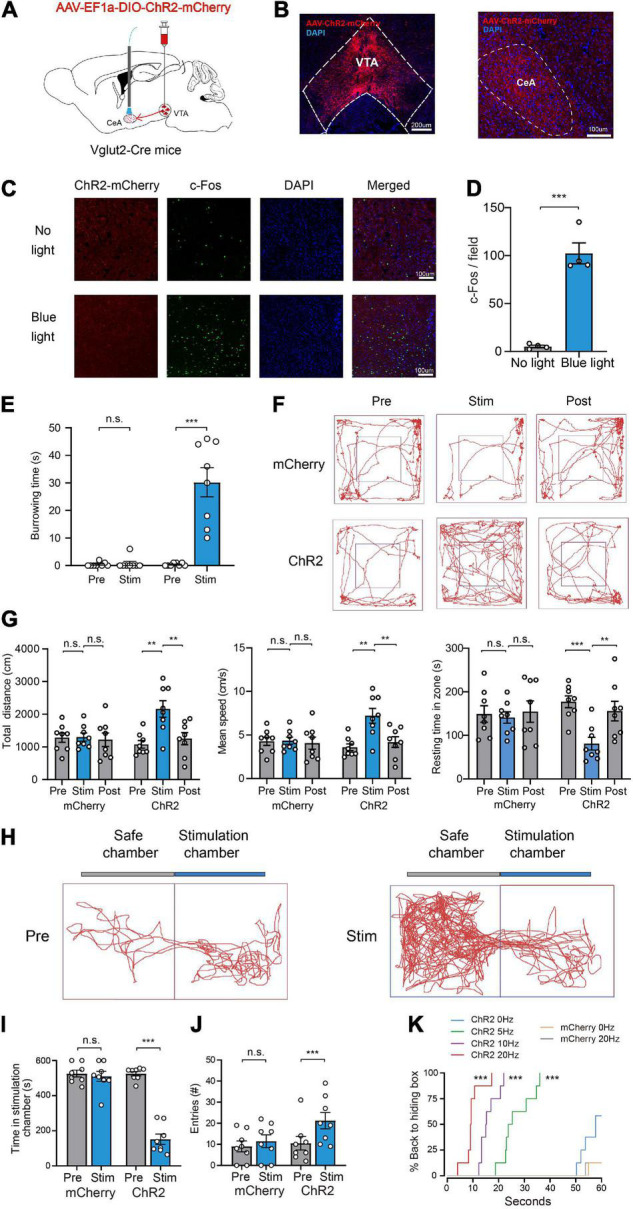
Photoactivation of glutamatergic VTA-CeA pathway promotes defensive behaviors. **(A)** Schematic of AAV-EF1a-DIO-ChR2-mCherry expression in the VTA and fiber implantation in the CeA for photostimulation. **(B)** Fluorescent images showing glutamatergic ChR2-mCherry SOMA in the VTA (left) and terminals projecting to the CeA (right). Scale bar represents 200 and 100 μm. **(C,D)** c-Fos expression in the CeA without (top) or with (bottom) light stimulation. (*n* = 4, 8 sections for each group. two-tailed unpaired *t*-test; *t*_6_ = 8.817, *P* < 0.001). Scale bars represent 100 μm. **(E)** Burrowing duration upon stimulation of the glutamatergic VTA-CeA pathway in the home cage [*n* = 8 ChR2 vs. *n* = 8 mCherry; two-way repeated-measures ANOVA, virus × stimulation, *F*(1,14) = 31.26, *P* < 0.001, Bonferroni *post hoc* comparison]. **(F)** Examples of tracking traces of mice in open field tests. **(G)** Photostimulation of the glutamatergic VTA-CeA pathway influenced total distance, mean speed and resting time in open field experiments [*n* = 8 ChR2 vs. *n* = 8 mCherry; two-way repeated-measures ANOVA, virus × stimulation, *F*(2,28) = 6.727, *P* = 0.0041, *F*(2,28) = 6.716, *P* = 0.0041, *F*(2,28) = 4.442, *P* = 0.0211, Bonferroni *post hoc* comparison]. **(H)** Example trajectories of mice in RTPP tests. **(I,J)** Photostimulation of glutamatergic VTA-CeA pathway influenced the stimulation chamber staying time and safe chamber entries of mice in the RTPP experiment. [*n* = 8 ChR2 vs. *n* = 8 mCherry; two-way repeated-measures ANOVA, virus × stimulation, *F*(1,14) = 82.24, *P* < 0.001, *F*(1,14) = 5.211, *P* < 0.05, Bonferroni *post hoc* comparison]. **(K)** Real-time probability of hiding upon stimulating the glutamatergic VTA-CeA pathway (*n* = 8 ChR2, *n* = 8 mCherry; Kaplan–Meier survival analysis with Bonferroni’s multiple comparison; Log rank = 99.124, *P* < 0.001, mCherry: 20 Hz-sham, *P* = 0.96; ChR2: 5 Hz-sham, *P* < 0.001; 10 Hz-sham, *P* < 0.001; 20 Hz-sham, *P* < 0.001). Data are expressed as mean ± SEM. **P < 0.01, ***P < 0.001, n.s., not significant.

Considering that the CeA is a key brain structure controlling fear responses (LeDoux, 2000; Isosaka et al., 2015) and has recently been implicated in innate defensive behaviors (Fadok et al., 2018; Terburg et al., 2018), we speculated that the CeA may participate in the defense-promoting effects of VTA glutamatergic neurons. To test this hypothesis, we tested the effects of optogenetic activation of the glutamatergic VTA-CeA pathway on defensive behaviors. To accomplish this, AAV-EF1a-DIO-ChR2-mCherry was microinjected into the VTA of Vglut2-Cre mice and optical fibers were bilaterally implanted above the CeA (Figures 4A,B). Immunofluorescence analysis revealed that blue light significantly increased c-Fos expression in the CeA of ChR2 group mice (Figures 4C,D).

Our behavioral results showed that, similar with glutamatergic VTA activation, photostimulation of the glutamatergic VTA-CeA pathway induced defense behaviors under various contexts. In the home-cage test, ChR2 group mice showed continuing burrowing behavior during the light stimulation period, seeming to make a refuge to hide from potential predators [mCherry group: Pre 0.4 ± 0.3 s vs. Stim 0.8 ± 0.8 s, ChR2 group: Pre 0.4 ± 0.2 s vs. Stim 30.3 ± 5.3 s, *n* = 8, two-way repeated-measures ANOVA, *F*(1,14) = 31.26, *P* < 0.001, Figure 4E]. In the open field test, light stimulation significantly increased the mean velocity [mCherry group: Pre 4.3 ± 0.5 cm/s vs. Stim 4.3 ± 0.4 cm/s vs. Post 4.1 ± 0.7 cm/s, ChR2 group: Pre 3.6 ± 0.4 cm/s vs. Stim 7.2 ± 0.8 cm/s vs. Post 4.2 ± 0.6 cm/s, *n* = 8, two-way repeated-measures ANOVA, *F*(2,28) = 6.727, *P* = 0.0041], total distance [mCherry group: Pre 1283.6 ± 144.9 cm vs. Stim 1303.1 ± 124.0 cm vs. Post 1220.1 ± 208.0 cm, ChR2 group: Pre 1077.3 ± 116.9 cm vs. Stim 2160.8 ± 253.2 cm vs. Post 1254.9 ± 185.0 cm, *n* = 8, two-way repeated-measures ANOVA, *F*(2,28) = 6.716, *P* = 0.0041] and decreased resting time in zone [mCherry group: Pre 149.2 ± 19.0 s vs. Stim 141.1 ± 13.1 s vs. Post 154.5 ± 24.5 s, ChR2 group: Pre 177.1 ± 13.5 s vs. Stim 80.8 ± 14.5 s vs. Post 155.8 ± 22.5 s, *n* = 8, two-way repeated-measures ANOVA, *F*(2,28) = 4.442, *P* = 0.0211] in the ChR2 group, but not the mCherry group (Figures 4F,G). In the RTPP test, light stimulation significantly decreased the time spent in the stimulation chamber and increased the number of entries into the safe chamber [Time in stimulation chamber, mCherry group: Pre 525.4 ± 19.5 s vs. Stim 510.0 ± 28.3 s, ChR2 group: Pre 525.0 ± 12.4 s vs. Sim 151.5 ± 29.1 s, *n* = 8, two-way repeated-measures ANOVA, *F*(1,14) = 82.24, *P* < 0.001; entries number, mCherry group: Pre 9.0 ± 2.5 vs. Stim 11.5 ± 3.0, ChR2 group: Pre 10.5 ± 3.2 vs. Stim 21.3 ± 3.9, *n* = 8, two-way repeated-measures ANOVA, *F*(1,14) = 5.211, *P* < 0.05] in the ChR2 group, but not the mCherry group (Figures 4H–J). In the hiding box test, light stimulation significantly increased the percentage of mice backing into the hiding box and decreased the hiding latency (Figure 4K). Collectively, these results illustrated that the CeA mediates the defense-promoting effect of the glutamatergic VTA.

### Photoactivation of the Glutamatergic VTA-CeA Pathway Initiates and Maintains Wakefulness

**FIGURE 5:**
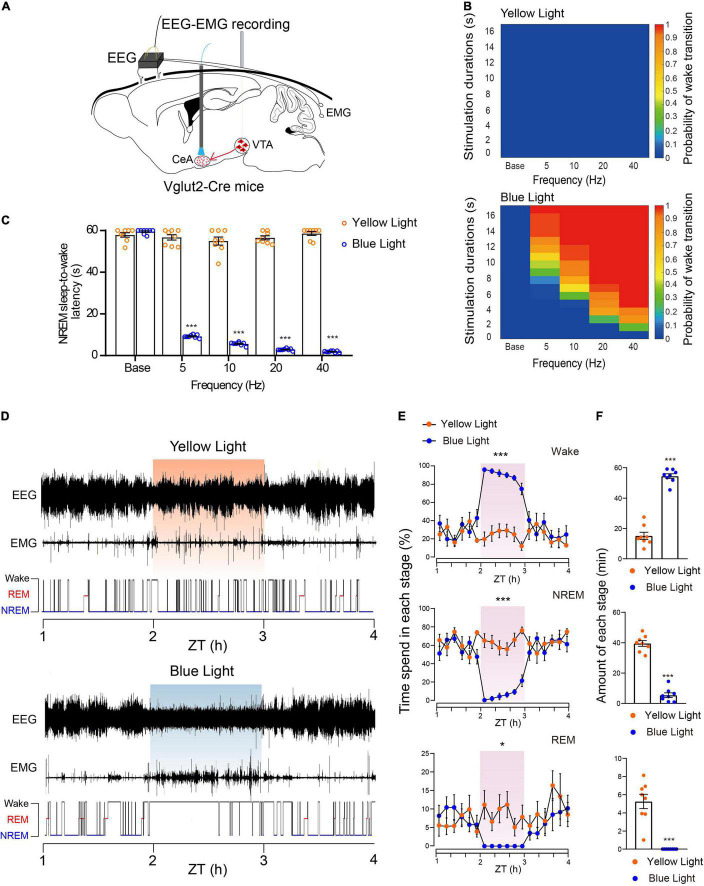
Photoactivation of the glutamatergic VTA-CeA pathway initiates and maintains wakefulness. **(A)** Diagram of *in vivo* photostimulation and EEG and EMG recording. **(B)** Heat maps showing the probability of wake transition upon yellow (top) or blue (bottom) light photostimulation during NREM sleep. **(C)** Latencies of NREM-Wake transitions under photostimulation at different frequencies (*n* = 8 per group, paired *t*-test; base, *t*_7_ = 1.517, *P* = 0.1730; 5 Hz, *t*_7_ = 32.96, *P* < 0.001; 10 Hz, *t*_7_ = 24.58, *P* < 0.001; 20 Hz, *t*_7_ = 53.77, *P* < 0.001; 40 Hz, *t*_7_ = 60.33, *P* < 0.001). **(D)** Example of EEG/EMG traces showing the effect of light stimulation of the glutamatergic VTA-CeA pathway on sleep–wake behaviors. **(E)** Time course change of sleep–wake behaviors during long-term photostimulation of the glutamatergic VTA-CeA pathway during ZT 02:00–03:00 [*n* = 8, two-way repeated-measures ANOVA; wake *F*(1,14) = 103.0, *P* < 0.001; NREM *F*(1,14) = 78.63, *P* < 0.001; REM *F*(1,14) = 8.604, *P* = 0.0109]. **(F)** Sleep and wake amount during ZT 02:00–03:00 light stimulation (*n* = 8, paired *t*-test; Wake *t*_7_ = 22.77, *P* < 0.001; NREM *t*_7_ = 27.37, *P* < 0.001; REM *t*_7_ = 6.615, *P* = 0.0003). Data are expressed as mean ± SEM. *P < 0.05, ***P < 0.001.

Because activation of the glutamatergic VTA-CeA pathway induces multiple defensive behaviors that depend on high arousal, we assumed that the VTA-CeA pathway was involved in sleep–wake behavior. To elucidate the role of the glutamatergic VTA-CeA pathway in sleep–wake regulation, AAV-EF1a-DIO-ChR2-mCherry was injected into the VTA and a fiber optic was implanted above the CeA to stimulate terminals from the glutamatergic VTA (Figure 5A).

Similar to the above optogenetic experiments, we first used a short-term stimulus paradigm. Our results showed that acute blue light stimulation of VTA terminals in the CeA induced instant NREM-to-wakefulness transition (Supplementary Video 6). Photostimulation of glutamatergic VTA-CeA pathway at 20 Hz instantly changed brain state and increased the probability of wakefulness (Supplementary Figure 2). Blue-light photostimulation frequency-dependently increased the probability of NREM-to-wakefulness transition and decreased the transition latency under different light stimulation frequency (Base, 57.9 ± 1.1 s at yellow light stimulation vs. 59.5 ± 0.4 s at blue light stimulation, *n* = 8, *P* = 0.1730; 5 Hz, 56.7 ± 1.3 s at yellow light stimulation vs. 9.2 ± 0.3 s at blue light stimulation, *n* = 8, *P* < 0.001; 10 Hz, 55.0 ± 2.0 s at yellow light stimulation vs. 5.6 ± 0.3 s at blue light stimulation, *n* = 8, *P* < 0.001; 20 Hz, 56.5 ± 1.0 s at yellow light stimulation vs. 2.9 ± 0.2 s at blue light stimulation, *n* = 8, *P* < 0.001; 40 Hz, 58.6 ± 0.9 s at yellow light stimulation vs. 1.9 ± 0.2 s at blue light stimulation, *n* = 8, *P* < 0.001, paired *t*-test, Figures 5B,C).

To further investigate whether activation of the glutamatergic VTA-CeA pathway is sufficient to maintain wakefulness, we adapted long-term photostimulation (10 ms, 20 Hz, 20 s-on, 40 s-off, 1 h) during the light period (Zeitgeber Time 02:00–03:00). Our results showed that similar to the effects of VTA cell-body activation, long-term photostimulation of terminals in the CeA produced sustaining wakefulness in mice (Figure 5D), significantly increased the amount of wakefulness, and concomitantly decreased the amount of NREM sleep and REM sleep (Figures 5E,F). The control yellow light had no effect on probability or latency of NREM sleep-to-wakefulness transition, and failed to change the wakefulness duration (wake, 15.2 ± 2.3 min at yellow light stimulation vs. 54.6 ± 1.6 min at blue light stimulation, *n* = 8, *P* < 0.001; NREM, 39.6 ± 1.9 min at yellow light stimulation vs. 5.4 ± 1.6 min at blue light stimulation, *n* = 8, *P* < 0.001; REM, 5.3 ± 0.8 min at yellow light stimulation vs. 0.0 ± 0.0 min at blue light stimulation, *n* = 8, *P* < 0.001, paired *t*-test, Figures 5E,F). Together, these results clearly illustrate that the CeA mediates the wakefulness-promoting effects of the glutamatergic VTA.

## Discussion

Previous studies have shown that glutamatergic neurons in the VTA, which project to the nucleus accumbens and the lateral hypothalamus, are involved in arousal regulation (Yu et al., 2019). In the current study, we found that activating the glutamatergic VTA-CeA pathway also strongly promotes arousal. These results suggest that glutamatergic VTA plays an important role in arousal promotion by transmitting signals to multiple downstream targets, including the CeA. *In vivo* electrophysiological analyses demonstrated that most of the sleep–wake-related neurons in the CeA fired slowly during NREM, but increased during wakefulness (Jha et al., 2005). Functional lesion of the CeA reduces wakefulness of rats, with shortened NREM latency and decreased locomotion in an arousing environment (Tang et al., 2005). Interestingly, previous studies have shown that some CeA neurons, such as the neurotensin (NTS)-expressing CeA neurons, function as sleep-promoting neurons (Kana et al., 2019). Kana et al. (2019) showed that CeA-NTS GABAergic neurons extensively inhibit arousal neurons and promote NREM sleep. Knocking down NTS expression in the CeA using CRISPR/Cas9 greatly reduced the magnitude of NREM increase induced by activation of CeA NTS neurons (Kana et al., 2019). It is important to note that the CeA is a complex nucleus that contains a variety of neurons with different molecular markers, projection targets and functional characteristics. Neurotensin-expressing neurons comprise only a fraction of the CeA neurons. Several subtypes of GABAergic neurons that have different molecular markers, projecting targets and functions coexist in the CeA. Different subtypes of CeA neurons may play different roles in the regulation of sleep–wake behavior. It is reasonable to infer that the some non-neurotensin-expressing neurons in the CeA promote arousal in the regulation of sleep–wake behavior. However, the specific types of CeA neurons that play a role in regulation of arousal have yet to be elucidated.

In addition to regulation of sleep–wake behavior, the VTA also plays a role in controlling motivational behaviors (Morales and Margolis, 2017). The VTA dopamine neurons are known to encode reward signals, while VTA glutamatergic neurons have been reported to regulate aversive emotions (Root et al., 2018). Optogenetic activation of glutamatergic fibers from VTA to the nucleus accumbens and the lateral habenula strongly drives conditioned place aversion (Root et al., 2014; Qi et al., 2016). The results of the current study showed that photoactivation of the glutamatergic VTA-CeA pathway drives strong escape and avoidance behavior, which is usually accompanied by aversive emotion. Recently, Barbano et al. (2020) demonstrated that VTA glutamatergic neurons play important roles in regulation of innate defensive behavior. The glutamatergic lateral hypothalamus (LH) transmits threat information to VTA glutamatergic neurons (Barbano et al., 2020). When the glutamatergic LH-VTA pathway is activated, animals show evasion behavior with a significantly increased number of runs and speeds to face the looming stimulus (Barbano et al., 2020). Consistent with the behavioral changes induced by activation of the glutamatergic LH-VTA pathway, our results showed that photoactivation of the glutamatergic VTA-CeA pathway strongly promotes defensive behaviors. These findings illustrate that the glutamatergic VTA is a pivotal relay of defensive behaviors, and that it may mediate defensive behaviors through the LH-VTA-CeA pathway.

The results of the present study also showed that activation of the glutamatergic VTA-CeA pathway induces defensive behavior. Notably, the GABAergic VTA-CeA pathway is also involved in the regulation of defense behavior. VTA GABAergic neurons receive direct excitatory inputs from the superior colliculus (Beier et al., 2015; Morales and Margolis, 2017). Previous studies showed that VTA GABAergic neurons play a pivotal role in response to a looming stimulus. Inhibition of VTA GABAergic neurons reduced defensive flight behavior, while their optogenetic activation resulted in defensive flight behavior (Zhou et al., 2019). Interestingly, the parallel VTA-CeA pathway mediates similar defensive behavior. A possible explanation for this might be that the glutamatergic and GABAergic neurons of VTA innervate different neuronal populations in the CeA. Neurotomical results showed that the CeA contains a variety of neuronal populations, including somatostatin-positive (SOM+) neurons and neurons that express protein kinase Cδ (PKCδ+) (Yu et al., 2016; van den Burg and Hegoburu, 2020). SOM+ neurons in the CeA have previously been shown to participate in passive defense behaviors that lead to the freezing of mice (Li et al., 2013; Penzo et al., 2014). Moreover, Li et al. (2013) found that SOM+ neurons were related to freezing through *in vivo* optogenetic manipulation. Optogenetic activation of SOM+ neurons induced robust freezing in mice, which subsequently disappeared upon the cessation of light (Li et al., 2013). Similarly, PKCδ+ neurons in the CeA are involved in the regulation of defense behavior. In contrast to SOM+ neurons in the CeA, CeA PKCδ+ neurons are known to mediate active defensive responses, leading to flight of mice (van den Burg and Hegoburu, 2020). Silence of CeA PKCδ+ neurons by pharmacogenetics significantly increased the degree of freezing in mice (Haubensak et al., 2010), and previous research showed that CeA neuronal circuit activity is tightly regulated by local inhibitory interactions (Day et al., 2005; Haubensak et al., 2010; Hou et al., 2016; Wood et al., 2016). CeA SOM+ neurons and PKCδ+ neurons have been shown to form reciprocal inhibitory connections that regulate each other’s activity (Fadok et al., 2018). Moreover, it has been suggested that PKCδ+ neurons inhibit freezing behavior through inhibition of SOM+ neurons when animals need to take active defense under fear stimulation (van den Burg and Stoop, 2019; van den Burg and Hegoburu, 2020). Therefore, activation of CeA flight neurons by VTA glutamatergic neurons and inhibition of CeA freezing neurons by VTA GABAergic neurons may be the mechanism underlying regulation of active defensive behavior by the parallel VTA-CeA pathway.

In addition to excessive defensive behaviors, social avoidance is another core feature of autism spectrum disorders (ASD) (Roberts et al., 2019). The VTA has been highly implicated in social behavior regulation (Gunaydin et al., 2014; Krishnan et al., 2017). For example, the sociability deficits induced by alterations of the ASD-related gene, Ube3a, have been shown to be associated with VTA glutamatergic neurons (Krishnan et al., 2017). Inhibition of VTA dopamine neurons decreases the exploration of non-familiar conspecifics by mice (Gunaydin et al., 2014; Bariselli et al., 2018). Given that the activity of VTA dopamine neurons is directly suppressed by local GABAergic neurons, which may be innervated by neighboring glutamatergic neurons (Dobi et al., 2010; Tan et al., 2012), it is possible that over-excitation of VTA glutamatergic neurons may lead to over-activation of GABAergic neurons and subsequent over-inhibition of VTA dopamine neurons. This may partly account for the impaired social communication that occurs in ASD. Moreover, the social impairment associated with ASD may be due to the inability to effectively differentiate between safety contexts and threat cues (Top et al., 2016). Threatening information is processed in the amygdala, which is a key brain region controlling behavioral and physiological fear responses (Isosaka et al., 2015; Jhang et al., 2018). As the main input nucleus in the amygdala, the BLA receives sensory information from the thalamus and cortex and sends abundant glutamatergic projects to the CeA, which is the major output nucleus of the amygdala (Duvarci and Pare, 2014). Abnormal processing of threatening information in the amygdala has been highly implicated in the generation of excessive fears observed in ASD (Suvrathan and Chattarji, 2011; Schoch et al., 2017). Additionally, the excitability of BLA glutamatergic neurons has been shown to be enhanced by deficiency of GABAergic neurotransmission within the BLA in many rodent models of ASD (Markram et al., 2008; Olmos-Serrano et al., 2010; Martin et al., 2014). In addition to the BLA, the VTA is another glutamatergic source of the CeA (Hnasko et al., 2012; Taylor et al., 2014). Our results showed that the activation of CeA by photoactivation of the glutamatergic VTA-CeA pathway elicits strong avoidance and other defensive behaviors in unthreatening contexts. It is possible that overactivity of the glutamatergic VTA-CeA pathway underlies the social avoidance that occurs in ASD, and suppression of this pathway may offer a feasible therapeutic strategy for this disease.

There are some limitations in the current study. As we have showed in the results, the number of fos-positive cells is much more than mcherry-positive cells. Although previous studies have reported that VTA glutamatergic innervated VTA dopaminergic neurons (Morales and Margolis, 2017), but it can’t rule out the possibility that VTA glutamate also innervates local GABAergic neurons. It is also reasonable to assume that activation of VTA glutamatergic triggers defensive behaviors through activating VTA GABAergic neurons. To further certificate the microcircuit between VTA glutamatergic and GABAergic neurons, *in vivo* electrophysiological study is need to carried out. Moreover, to more clearly elucidate the causal relationship between the glutamatergic VTA-CeA pathway and arousal and defensive behaviors, further experiments of optogenetic or chemogenetic suppression are need to be conducted.

## Data Availability Statement

The original contributions presented in the study are included in the article/Supplementary Material, further inquiries can be directed to the corresponding author/s.

## Ethics Statement

The animal study was reviewed and approved by the Ethics Committee of Laboratory Animal Management of Fujian Medical University. All of the experimental procedures were carried out in accordance with the principles of China Regulations on the Administration of Laboratory Animals, the Decree No. 2 of National Science and Technology Commission of the People’s Republic of China.

## Author Contributions

S-YC, JY, and Y-DH: conceptualization, experimental design, investigation, and writing the original draft. H-YC: methodology and investigation. P-CL: investigation and funding acquisition. W-FW, Y-HZ, C-WZ, S-XZ, Y-PL, L-YY, Z-XH, K-QH, Z-TL, and Y-HH: investigation and data curation. PC: conceptualization, project administration, and funding acquisition. LC and S-YW: experimental design, supervision, funding acquisition, resources, and writing (review and editing). All authors contributed to the article and approved the submitted version.

## Conflict of Interest

The authors declare that the research was conducted in the absence of any commercial or financial relationships that could be construed as a potential conflict of interest.

## Publisher’s Note

All claims expressed in this article are solely those of the authors and do not necessarily represent those of their affiliated organizations, or those of the publisher, the editors and the reviewers. Any product that may be evaluated in this article, or claim that may be made by its manufacturer, is not guaranteed or endorsed by the publisher.
