# Supplementary material for: Control of Behavioral Arousal and Defense by a Glutamatergic Midbrain-Amygdala Pathway in Mice
Source: Front Neurosci. 2022 Apr 18;16:850193. doi: 10.3389/fnins.2022.850193 (PMC9070111; doi:10.3389/fnins.2022.850193)
Supplement: Supplementary file 1 [file Image_1.pdf]

Supplementary Material

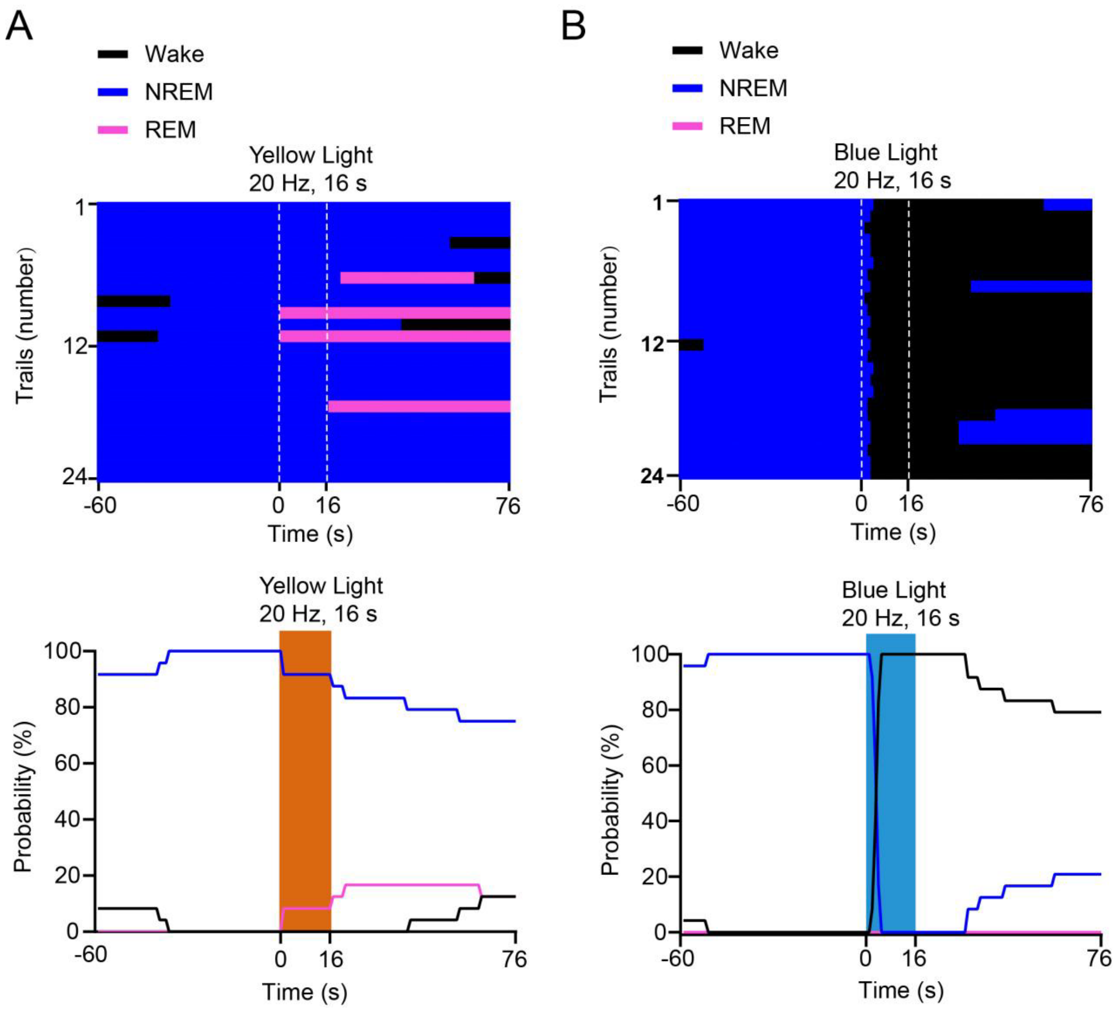

Figure S1. Photoactivation of VTA glutamatergic neurons changes brain states and increases the probability of wakefulness.

A. Brain status of all trails before, during and after 20 Hz yellow light stimulation of VTA glutamatergic neurons in ChR2 mice (top); Probability of Wake, NREM and REM status before, during and after 20 Hz yellow light stimulation of VTA glutamatergic neurons in ChR2 mice (bottom).

B. Brain status of all trails before, during and after 20 Hz blue light stimulation of VTA glutamatergic neurons in ChR2 mice (top); Probability of Wake, NREM and REM status before, during and after 20 Hz blue light stimulation of VTA glutamatergic neurons in ChR2 mice(bottom).

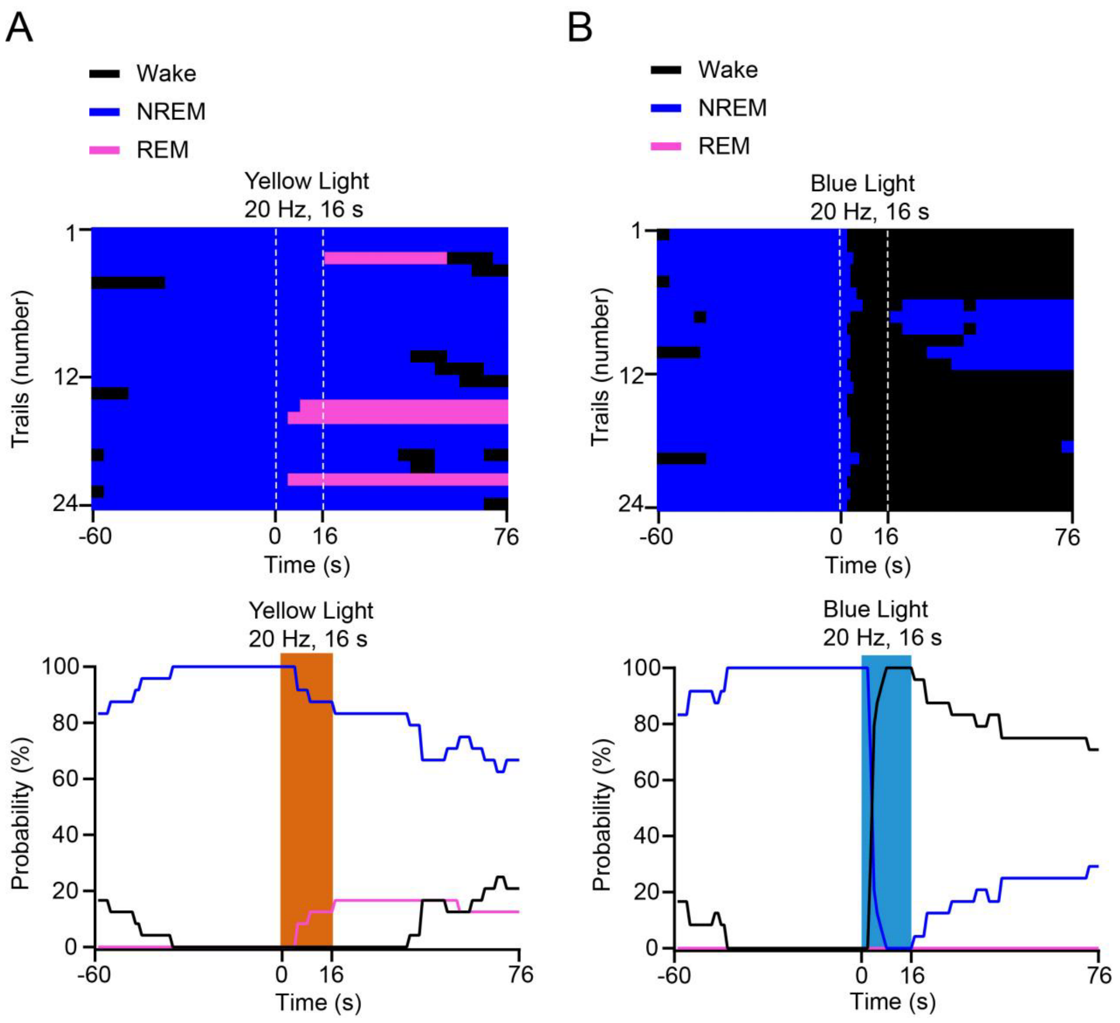

Figure S2. Photoactivation of glutamatergic VTA-CeA pathway changes brain states and increases the probability of wakefulness.

A. Brain status of all trails before, during and after 20 Hz yellow light stimulation of glutamatergic VTA-CeA pathway in ChR2 mice (top); Probability of Wake, NREM and REM status before, during and after 20 Hz yellow light stimulation of glutamatergic VTA-CeA pathway in ChR2 mice (bottom).

B. Brain status of all trails before, during and after 20 Hz blue light stimulation of glutamatergic VTA-CeA pathway in ChR2 mice (top); Probability of Wake, NREM and REM status before, during and after 20 Hz blue light stimulation of glutamatergic VTA-CeA pathway in ChR2 mice (bottom).
